# Novel Genomic Regions Associated with Intramuscular Fatty Acid Composition in Rabbits

**DOI:** 10.3390/ani10112090

**Published:** 2020-11-11

**Authors:** Houda Laghouaouta, Bolívar Samuel Sosa-Madrid, Agostina Zubiri-Gaitán, Pilar Hernández, Agustín Blasco

**Affiliations:** Institute for Animal Science and Technology, Universitat Politècnica de València, 46022 Valencia, Spain; houlag@posgrado.upv.es (H.L.); bosomad@posgrado.upv.es (B.S.S.-M.); agzugai1@posgrado.upv.es (A.Z.-G.); phernan@dca.upv.es (P.H.)

**Keywords:** intramuscular fat, fatty acids, divergent selection, genome-wide association study, rabbits

## Abstract

**Simple Summary:**

A divergent selection experiment on intramuscular fat (IMF) content was carried out during nine generations in rabbits. The IMF content was successfully improved through generations. Besides, selection for IMF content generated a correlated response on its composition. Association analyses were performed to understand the genetic background of IMF composition using two rabbit lines divergently selected for IMF content. Several genomic regions and genes were identified, revealing the polygenic nature of the intramuscular fatty acid composition in rabbits.

**Abstract:**

Intramuscular fat (IMF) content and its composition affect the quality of meat. Selection for IMF generated a correlated response on its fatty acid composition. The increase of IMF content is associated with an increase of its saturated (SFA) and monounsaturated (MUFA) fatty acids, and consequently a decrease of polyunsaturated fatty acids (PUFA). We carried out a genome wide association study (GWAS) for IMF composition on two rabbit lines divergently selected for IMF content, using a Bayes B procedure. Association analyses were performed using 475 individuals and 90,235 Single Nucleotide Polymorphisms (SNPs). The main objectives were to identify genomic regions associated with the IMF composition and to generate a list of candidate genes. Genomic regions associated with the intramuscular fatty acid composition were spread across different rabbit chromosomes (OCU). An important region at 34.0–37.9 Mb on OCU1 was associated with C14:0, C16:0, SFA, and C18:2n6, explaining 3.5%, 11.2%, 11.3%, and 3.2% of the genomic variance, respectively. Another relevant genomic region was found to be associated at 46.0–48.9 Mb on OCU18, explaining up to 8% of the genomic variance of MUFA/SFA. The associated regions harbor several genes related to lipid metabolism, such as *SCD*, *PLIN2*, and *ERLIN1.* The main genomic regions associated with the fatty acids were not previously associated with IMF content in rabbits. Nonetheless, *MTMR2* is the only gene that was associated with both the IMF content and composition in rabbits. Our study highlighted the polygenic nature of the fatty acids in rabbits and elucidated its genetic background.

## 1. Introduction

Intramuscular fat (IMF) content and its fatty acid composition are key traits influencing meat quality [1]. The increase of IMF improves the juiciness, tenderness, and flavor of the meat [2]. IMF content and its fatty acid composition determine also the nutritional value of the meat. Individual saturated (SFA) and monounsaturated (MUFA) fatty acids could be biosynthesized. However, linoleic (precursor of n-6 fatty acids) and alpha linoleic (precursor of n-3 fatty acids) are both polyunsaturated fatty acids (PUFA) that cannot be biosynthesized, thus they must be obtained from the diet [3]. High intake of SFA increases low-density lipoprotein (LDL) cholesterol which is associated with cardiovascular diseases. In contrast, PUFA decrease LDL cholesterol [4]. Meat consumers are interested in the fatty acid composition of the meat, demanding healthy products rich in PUFA respect to SFA. 

The high heritability of IMF and its substantial variability allowed its improvement through selection in cattle [5], chickens [6,7], and pigs [8]. In rabbits, a divergent selection experiment on IMF was carried out at the Universitat Politècnica de València during nine generations revealing a correlated response on its fatty acid composition [9,10]. The high-IMF line had greater percentages of MUFA than the low-IMF line. In contrast, the low-IMF line had greater percentages of PUFA [9].

Several association analyses were performed in order to unravel the genetic background of the IMF fatty acid composition. Previous Genome-Wide Association Studies (GWAS) reported genetic markers and genes associated with fatty acid composition in pigs [11,12,13] and cattle [14,15,16]. The main candidate genes were Stearoyl-CoA desaturase-1 (*SCD*), Leptin receptor (*LEPR*), Fatty Acid Synthase (*FASN*), and Fatty Acid Elongase (*ELOVL*). These studies underlined the complexity and the polygenic nature of the fatty acids in pigs [11,12,13] and cattle [14,15,16]. To our knowledge, there are no previous association analyses for the intramuscular fatty acid composition in rabbits. A previous study identified four genomic regions related to IMF content in rabbits, using two rabbit lines divergently selected for IMF [10]. IMF content and its fatty acid composition can share genomic regions with pleiotropic effects, since they have moderate to high genetic correlations [9]. In this study, we carried out a GWAS for intramuscular fatty acid composition, using the same previous rabbit lines divergently selected for IMF, in order to identify genomic regions associated with this trait and to detect putative candidate genes.

## 2. Materials and Methods 

### 2.1. Animals

The present study was carried out on 478 individuals from the 9th generation of two lines divergently selected for IMF (239 rabbits each line). The base population had 13 sires and 83 does [17]. High (high-IMF) and low (low-IMF) lines had approximately 8 sires and 40 does per generation within each line, from the 1st to the 7th generation [9]. The latest generations (8th and 9th) comprised approximately 10 sires and 60 does per generation for each line [10]. Rabbits were weaned at 4 weeks of age and housed collectively until slaughter. Both lines were contemporarily reared in the same building having the same management and fed ad libitum with a standard commercial diet, containing 15.1% of crude protein, 14.5% of crude fiber and 2.8% of fat. The housing ventilation was controlled, and the photoperiod was constant (16:8 h). Two full sibs of the first parity of each doe were slaughtered at 9 weeks of age by exsanguination prior electrical stunning. Carcasses were chilled for 24 h at 4 °C. From each animal, Longissimus thoracis et lumborum muscle (LM) was excised and freeze-dried. IMF content was measured in LM by near-infrared reflectance spectroscopy (NIRS) and expressed in g/100 g of LM [18]. Twenty percent of the samples were chemically analyzed to test NIRS results. More details of the experiment can be found in previous works [9,17,18]. 

### 2.2. Phenotypes

Fatty acids were quantified by gas chromatography. First, we prepared fatty acid methyl esters (FAME) [19]. Further, FAME were analyzed by gas chromatography (FOCUS, Thermo, Milan, Italy), using a split/splitless injection, flame ionization detection, and a fused silica capillary column SPTM 2560 (Supelco, Bellefonte, PA, USA) (100 m × 0.25 mm × 0.2 μm film thickness). We injected FAME using a split ratio of 1/100 and Helium as a carrier gas (20 cm/s). The oven temperature was set at 140 °C during 5 min, then increased (4 °C/min) to 240 °C and maintained at 240 °C during 30 min. Detector and injector were at a temperature of 260 °C. We determined the fatty acids by comparing their retention times with the standards supplied by Supelco (Bellefonte, PA, USA), after that individual fatty acids were quantified using C13:0 as internal standard. The fatty acids contents were expressed as percentage of total fatty acids. 

The studied traits were the main individual fatty acids C14:0, C16:0, C18:0, C16:1n7, C18:1n9, C18:2n6, C18:3n3, C20:4n6, the groups SFA, MUFA, PUFA, and the ratios PUFA/SFA, MUFA/SFA, n-6/n-3. Other minor individual fatty acids such as C12:0, C15:0, C17:0, C20:0, C18:1n7, C20:1n11, C22:1n9, C18:3n6, C20:2n6, C20:3n6, C20:3n3, C20:5n3, C22:4n6, C22:5n3, and C22:6n3 were not studied individually, however, they were considered to estimate SFA, MUFA, and PUFA groups. The studied fatty acids accounted for more than 93% of total fatty acids.

### 2.3. Genotypes and Quality Control

Rabbits were genotyped using the Affymetrix Axiom OrcunSNP Array (Affymetrix Inc., Santa Clara, CA, USA). The Single Nucleotide Polymorphisms (SNP) array contained 199,692 molecular markers. Quality control was performed using Axiom Analysis Suite v.4.0.3 of Thermo Fisher Scientific (Santa Clara, CA, USA). SNPs with a call rate greater than 0.95, a minor allele frequency greater than 0.05, and a known chromosome position were retained for the association analyses. In addition, individuals with a missing genotype frequency greater than 0.05 were excluded from the dataset. The missing genotypes were imputed by Beagle software v.4.1 [20] using 96 ancestors. A score of *R*^2^ > 0.75 was used as quality parameter for imputed genotypes. After quality control, 475 animals (low-IMF: 236; high-IMF: 239) and 90,235 SNPs remained in the dataset.

### 2.4. Genome-Wide Association Study (GWAS) 

The association analyses were performed using Bayesian procedures [21]. A Multiple-Marker Regression was performed under the following Bayes B model as in [21,22]:

$$y=Xb+\sum_{j=1}^{k}z_{j}\alpha_{j}\delta_{j}+e$$

where ***y*** is the phenotypes vector. ***X*** is the incidence matrix for fixed effects and ***b*** is the vector of the fixed effects of month (five levels), sex (two levels), and parity order (three levels), with bounded flat prior density. ***z_j_*** is the genotypes vector for a SNP at locus *j* (*j* = 1, …, *k,* where *k* is the number of SNPs after quality control). *α_j_* is the random substitution effect for SNP *j*, distributed as $t_{\nu }\left(0,\sigma_{\alpha }^{2}\right)$, where $\sigma_{\alpha }^{2}$ was derived as in [21,22] from the genetic variance of each trait estimated using data of the fatty acids from all generations of the divergent selection experiment for IMF [9]. *δ_j_* is a random 0/1 variable (*δ_j_* = 1 represents the presence of SNP *j* with probability 1−π and *δ_j_* = 0 represents the absence of SNP *j* with probability π). ***e*** is the residual term, distributed as $N\left(0,I\sigma_{e}^{2}\right)$, where $\sigma_{e}^{2}$ has a bounded flat prior density. It was assumed π = 0.9988, according to a previous GWAS for IMF in rabbits and to exploratory analyses for the studied traits [10]. Marginal posterior distributions of the model unknowns were estimated using Monte Carlo Markov chains. A total of 500,000 iterations were performed with a burn-in of 100,000 and a lag of 40. In this study, 1982 non-overlapping genomic windows of 1 Mb were a priori allocated to the 21 autosomes using the SNPs that remained after quality control. The windows have an average of 45 SNPs per 1 Mb window. The contributions of the windows to the genomic variance were computed as the posterior distribution of the genomic variance explained by the SNPs within each 1 Mb genomic window. All GWAS were performed using the GenSel software [22].

Bayes factors (BF) were estimated to evaluate the statistical relevance of the association between SNPs and traits. Bayes factors were calculated as follows [23]:

$$BF_{j}=\frac{\frac{\hat{p}j}{1-\hat{p}j}}{\frac{1-\pi }{\pi }}$$

where $\hat{p}j$ is the posterior probability of SNP *j*. 

### 2.5. Genomic Regions Associated with the Fatty Acid Composition

The genomic windows exceeding 1.0% of the genomic variance of the trait were considered to be associated with the trait. In addition, the genomic windows exceeding 0.5% of the genomic variance of the trait and having SNPs with a BF greater than 10 were also considered as associated with the trait. These thresholds of 1% and 0.5% represent 20 and 10 times the expected percentage of the genomic variance explained by each genomic window, respectively. SNPs with a BF greater than 10 were considered as associated with the trait [24]. The associated genomic windows were extended to ± 500 Kb from the first and last associated SNP in order to consider nearby associated SNPs, taking into account the linkage disequilibrium (LD). These new genomic windows are termed as “extended regions”.

### 2.6. Identification of Candidate Genes 

Genes were retrieved from the extended regions. Candidate genes were obtained from the Ensembl Genes 98 database using the *Oryctolagus cuniculus* as the reference genome [25]. The biological functions were retrieved from the Database for Annotation, Visualization, and Integrated Discovery (DAVID) v.6.8 [26]. The genes related to lipid metabolism have been also investigated using the gene ontology (GO) [27].

### 2.7. Linkage Disequilibrium Analysis

In addition, we examined the LD in the most relevant extended regions to visualize the relationships between the SNPs. LD analyses were performed using plink software [28] and LDheatmap function from R [29].

### 2.8. Internal Validation

#### 2.8.1. Permutation Test

Results should be validated in order to overcome the problem of multiple testing. The phenotypes were resampled 100,000 times to remove any possible relationship between the phenotypes and genotypes, using the following covariates: sex, month, parity order, and the three first components of the principal component analysis of the genotypes (explaining 36% of the variance) in order to avoid population structures. Permutation testing was performed on the new data corresponding to the null hypothesis. Empirical *p*-values (EMP1) were provided for each SNP. EMP1 was estimated as K/N, where N is the total number of permutations and K is the number of times the SNP comes out associated in the simulated data. SNPs with EMP1 values close to 10^−5^ were deemed true positives [30]. Permutation testing was performed using Plink software [28].

#### 2.8.2. Within line GWAS

GWAS within line were performed to compare the SNPs effects. Confidence intervals of the SNPs effects (effect ± 2SD) were calculated in both lines. SNPs with overlapping confidence intervals were considered as true positive associations. In contrast, SNPs with non-overlapping confidence intervals were discarded, since we assumed that these associations were caused by sampling effect and genetic drift.

### 2.9. Ethics Statement

All experimental procedures were approved by the Universitat Politècnica de València. Research Ethics Committee, according to Council Directives 98/58/EC and 2010/63/EU (reference number 2017/VSC/PEA/00212).

## 3. Results and Discussion

### 3.1. Descriptive Statistics of the Intramuscular Fatty Acid Composition

Table 1 shows descriptive statistics of the studied traits. The fatty acids profile of rabbit meat comprises higher percentages of SFA and PUFA (36.4% and 39.2%, respectively), while MUFA presented a lower percentage (24.4%). The most ubiquitous fatty acids were linoleic (C18:2n6), palmitic (C16:0), and oleic (C18:1n9) acids, representing 27.7%, 25.7% and 20.3% of total fatty acids, respectively. Ratios PUFA/SFA, MUFA/SFA, and n-6/n-3 were 1.08, 0.67, and 17.7, respectively. These results are in agreement with previous results in rabbits [9]. 

### 3.2. Genomic Data

The association analyses were performed using the 475 individuals and 90,235 SNPs remaining in the dataset after quality control. Allele frequencies differ among lines due to the random genetic drift and selection. As the animals came from a divergent selection experiment for IMF, genes with large causal variants will show extreme frequencies for alternate alleles between lines due to selection. We expect that high-IMF line will show genes with high frequencies given a reference allele, while the same genes will have lower frequencies in low-IMF line. Therefore, these genes will show intermediate frequencies when both lines are used. We did not consider the line as a fixed effect to take advantage from these extreme differences caused by selection and detect the genes with large causal variants. We also performed GWAS with the fixed effect of line in the model. The same associated genomic regions were obtained with lower percentages of the genomic variances of the traits. Divergent selection experiments can improve the detection power, according to simulation studies, especially for large causal variants and their nearby SNPs [31,32]. This statement is also certain for correlated traits. Thus, GWAS would detect in our experiment important genomic regions associated with the correlated traits, despite the small population size.

### 3.3. Genomic Regions Associated with the Intramuscular Fatty Acid Composition 

**Figure 1:**
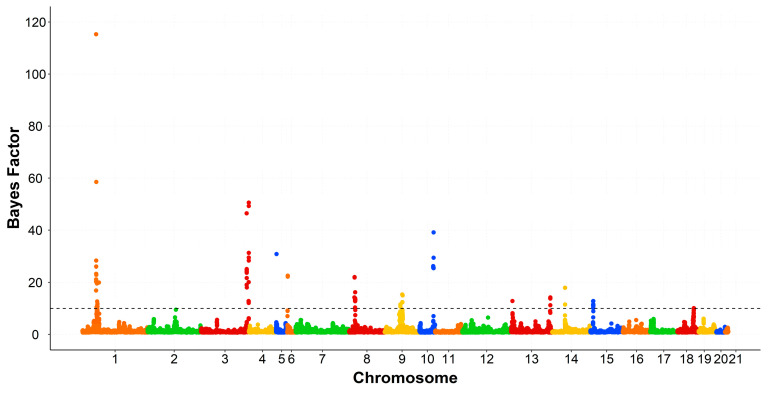
Manhattan plot of genome-wide association study for saturated fatty acids. The black dashed line indicates the Bayes factor threshold of 10. Each dot represents a Single Nucleotide Polymorphism (SNP). X-axis: chromosomes. Y-axis: Bayes factor.

**Figure 2:**
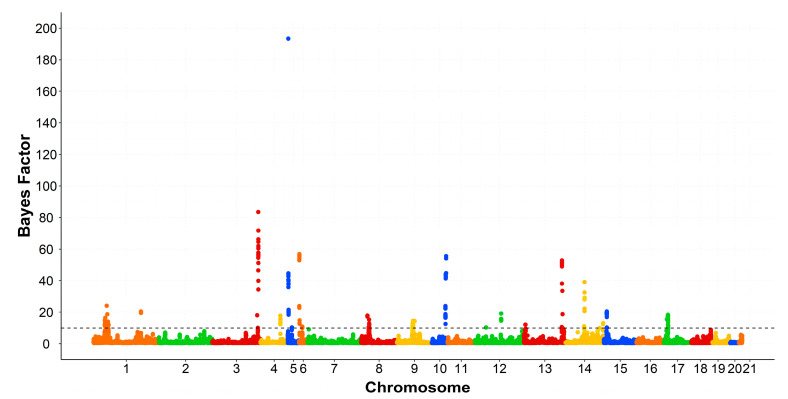
Manhattan plot of genome-wide association study for polyunsaturated fatty acids. The black dashed line indicates the Bayes factor threshold of 10. Each dot represents a SNP. X-axis: chromosomes. Y-axis: Bayes factor.

**Figure 3:**
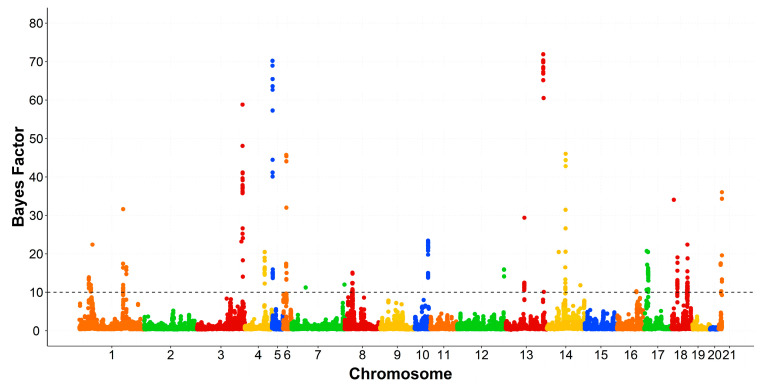
Manhattan plot of genome-wide association study for monounsaturated fatty acids. The black dashed line indicates the Bayes factor threshold of 10. Each dot represents a SNP. X-axis: chromosomes. Y-axis: Bayes factor.

The genomic windows that exceeded the thresholds levels were spread across several chromosomes. All rabbit chromosomes (OCU), except OCU7, OCU11, and OCU20. The associated regions with SFA, MUFA, PUFA, and the ratios were displayed in Table 2, Table 3, Table 4 and Table 5, respectively. The associated SNPs were tested using GWAS within line and permutation testing. The validated SNPs with a BF greater than 50 were presented in Table S1. Manhattan plots of SFA, PUFA, and MUFA groups were displayed in Figure 1, Figure 2 and Figure 3, respectively.

On OCU1, a large genomic region at 34.0–37.9 Mb was associated with C14:0, C16:0, C18:0, and SFA, explaining 3.54%, 11.2%, 0.87%, and 11.3% of the genomic variances, respectively (Table 2). The same region was associated with C18:2n6, C20:4n6, PUFA, PUFA/SFA, and n-6/n-3, explaining 3.18%, 1.57%, 2.41%, 2.98%, and 1.45% of the genomic variances, respectively. This genomic region harbors two genes related to lipids metabolism, alkaline ceramidase 2 (*ACER2*) and perilipin 2 *(PLIN2). ACER2* promotes the hydrolysis of ceramides, the generation of sphingosine, and consequently the biosynthesis of sphingolipids [33,34]. Besides, the protein encoded by *PLIN2* covers the intracellular lipid droplets and plays a major role in their stabilization [35]. In pigs, *PLIN2* was considered as a useful marker for lean growth and its expression was positively associated with IMF content [36,37]. In rabbits, a selection signature for IMF was located in this genomic region, and presented high values for cross population—composite likelihood ratio (XP-CLR) and cross population—extended haplotype homozygosity (XP-EHH) [38]. Hence, this region harbors genes of pleiotropic effect given its association with the IMF content and its composition. Moreover, this region disclosed two validated SNPs with high BF. The first SNP at position 34.70 Mb was highly associated with C16:0 (BF = 177) and SFA (BF = 115), while the second SNP at 35.18 Mb was associated with SFA (BF = 59) (Table S1, Figure 1). 

On the same chromosome (OCU1), the genomic window at 121.0–121.9 Mb was associated with C14:0, C18:0, C18:1n9, and MUFA. This region harbors the *MTMR2* gene involved in lipid metabolic processes according to DAVID database. *MTMR2* was also associated with the IMF content in rabbits, the genomic region at 120.6–121.9 Mb on OCU1 accounted for 2.03% of the genomic variance of IMF content [10]. Besides, *MTMR2* gene was located at a signature of selection for IMF in pigs [39].

The genomic region at 25.0–26.9 Mb on OCU3 accounted for 6.05% of the genomic variance of linoleic acid (C18:2n6). Two candidate genes were retrieved, *FGF1* and *NR3C1*. *FGF1* was also considered as a candidate gene for IMF in chickens [40], while an expression Genome-Wide Association Study (eGWAS) reported the implication of *NR3C1* gene in lipid metabolism in pigs [41]. On the same chromosome, the genomic region at 149–149.9 Mb was associated with almost all the studied traits except C16:1n7, C18:3n3, MUFA/SFA, and n-6/n-3. This region includes *ENSOCUG00000000157* novel gene related to lipid biosynthesis. The novel gene is also known as *ST3GAL1* in humans, pigs, and mice. The validated SNPs at this region showed high values of the BF (up to 141 for the ratio PUFA/SFA, 114 for C18:0 and 101 for C14:0) (Table S1). Moreover, these associations were corroborated by high values of the posterior probability of association (PPA). The PPA of C14:0, C16:0, and C18:0 were 0.96, 0.98, and 0.99, respectively (Table 2). 

The associated genomic region at 7.2–7.9 Mb on OCU5 did not map to genes related to lipid metabolism, however, it encompasses the highest BF. The validated SNP at position 7.43 Mb on OCU5 was associated with C16:0 (BF = 459), C16:1n7 (BF = 77), PUFA (BF = 193), and the ratio PUFA/SFA (BF = 390) (Table S1, Figure 2). 

Three different regions on OCU6 were associated with the fatty acid composition. The first region at the start of the chromosome was associated with C16:0, PUFA, and PUFA/SFA. However, it did not map to putative candidate genes related to lipid metabolism. The second genomic window at 5–5.9 Mb was associated with C18:2n6, and included two genes related to lipid metabolism (*LITAF* and *SNX29)*. *LITAF* regulates the response to lipopolysaccharide according to gene oncology (GO: 0032496), whereas *SNX29* affects phosphatidylinositol (family of lipids) binding (GO: 0035091). *SNX29* was also a candidate gene for IMF in pigs [42]. The third region at 8–9.9 Mb was associated with C16:1n7, C18:1n9, and MUFA. This region harbors two genes related to lipid metabolism (*SMG1* and *GDE1)* according to DAVID database. 

On OCU8, the genomic region at 21.0–21.9 Mb was associated with C18:0, C16:1n7, C18:1n9, MUFA, and PUFA. This region harbors *PIK3C2G*, *PLEKHA5*, and *PLCZ1* genes related to lipid metabolism. *PIK3C2G* affects phosphatidylinositol binding (GO: 0035091), while *PLEKHA5* controls lipid binding (GO: 0032266, GO: 0070273, and GO: 0010314). On the other hand, *PLCZ1* regulates lipid metabolic and catabolic processes (GO: 0006629 and GO: 0016042, respectively). On the same chromosome, the genomic region at 53–53.8 Mb was associated with MUFA/SFA. This region harbors *DGKH* gene involved in triglycerides degradation [43]. The genomic region at 55.3–55.9 Mb explained 4.77% of the genomic variance of C18:2n6, however, it did not contain putative candidate genes.

The genomic region at 12.0–12.5 Mb on OCU9 accounted for 1.05% of the genomic variance of C16:1n7. This region harbors *PPARG* gene involved in adipocyte differentiation [44]. *PPARG* was also associated with fat deposition in sheep [45]. In pigs, two polymorphisms in the transcriptional regulatory region of this gene were related to IMF content [46]. Moreover, a correlation (0.64) was detected between mRNA *PPARG* and IMF content in Korean cattle [47]. On the same chromosome, the region at 64–65.9 Mb was found to be associated with PUFA and PUFA/SFA. This region harbors *NPC1* and *OSBPL1* genes. *NPC1* is associated with lipid transport [48], while *OSBPL1* is involved in both lipid transport (GO: 0006869) and binding (GO: 0008289).

On OCU10, the genomic region at 5–5.6 Mb was associated with the ratio MUFA/SFA. This region harbors the *ITGB8* gene related to lipid metabolic process. This gene was also located at a signature of selection for IMF content in pigs [39].

Two genomic regions on OCU13 were found to be associated with the fatty acids. The first region at 33–34.9 Mb accounted for 3.42% of the genomic variance of linoleic acid (C18:2n6). This region includes five genes related to lipid metabolism according to DAVID database (*CRP*, *DCAF8*, *FCER1A, PIGM*, and *ATP1A2)*. The second region at 126.0–126.9 Mb was associated with C18:0, C18:1n9, and MUFA, harboring *HEYL*, *MFSD2A*, and *PPT1* genes. *MFSD2A* regulates the docosahexaenoic acid (C22:6n3) transport [49]. Validated associated SNPs at this region showed high values of the BF (96 for C18:0, 84 for C18:1n9, and 72 for MUFA; Table S1; Figure 3).

Stearic (C18:0) and oleic (C18:1n9) acids were associated with the genomic window at 82–82.9 Mb on OCU14. This region harbors the *ADIPOQ* gene that encodes for the adiponectin involved in fatty acids metabolism [50]. *ADIPOQ* was also associated with IMF in cattle [51]. Moreover, a previous study reported a high correlation between its expression and IMF in cattle [52]. On the same chromosome, the genomic region at 78.2–78.9 Mb was associated with the ratio MUFA/SFA, presenting *ATP11B* gene involved in phospholipid transport (GO: 0015914). 

The genomic region at 10–10.9 Mb on OCU15 was associated with C16:0, SFA, C18:2n6, PUFA, and the ratio PUFA/SFA. Two genes related to lipid metabolism mapped to this region (*LRAT* and *TLR2)*. On the same chromosome, the genomic region at 70–71.9 Mb accounted for 3.59% of the genomic variance of linoleic acid (C18:2n6), harboring *ANXA3* and *GK2* genes related to lipid metabolism.

Two regions on OCU16 were associated with the fatty acids. The first region at 62.0–62.9 Mb was associated with C18:2n6 and MUFA/SFA, harboring *HSD11B1* gene related to lipid metabolic process. The second region at 71–72.9 Mb was associated with C18:3n3 and n-6/n-3, explaining 1.67% and 1.04% of the genomic variances, respectively. This region harbors *ENSOCUG00000006240* novel gene known as *NR5A2* in humans. *NR5A2* regulates cholesterol homeostasis (GO: 0042632).

On OCU17, the region at 9–10.9 Mb explained 3.87% of the genomic variance of C18:2n6, however, it did not contain candidate genes. Besides, the region at 12–13.9 Mb was associated with C16:1n7, MUFA, C18:2n6, and PUFA. This genomic region harbors three genes related to lipid metabolism (*ALDH1A2*, *LIPC*, and *MYOE1)*. *LIPC* regulates lipid metabolic process (GO: 0006629), whereas *MYO1E* controls lipid binding (GO: 0035091). 

The genomic region at 11–11.9 Mb on OCU18 was associated with the ratio MUFA/SFA, harboring *SAMD8* gene related to ceramides biosynthetic process (GO: 0046513). Besides, a large genomic region at 46–48.9 Mb on OCU18 was associated with the majority of the traits, explaining 7.9% of the genomic variance of the ratio MUFA/SFA. This region harbors the following genes: *GOT1*, *ERLIN1*, *ENSOCUG00000001375*, and *ENSOCUG00000014801*. *ERLIN1* promotes lipid binding; it encodes for proteins that bind to cholesterol and interacts with the sterol regulatory element binding protein (SREBP). This latter stimulates the cholesterol and fatty acid synthesis in liver [53,54]. Moreover, a previous GWAS reported that *ERLIN1* was associated with lipid metabolism in cattle [55]. The novel genes *ENSOCUG00000001375* and *ENSOCUG00000014801* are known in pigs and humans as Stearoyl-CoA Desaturase (*SCD*). *SCD* gene is responsible for the biosynthesis of MUFA from SFA. A strong association between *SCD* and the fatty acid composition was reported in pigs [11,56], cattle [16], and goats [57]. In addition, an eGWAS study showed a high correlation (0.78) between *SCD* and *PPARG* expressions [58]. The latter gene was located on the associated genomic region at OCU9.

The genomic region at 21.0–21.9 Mb on OCU19 was associated with linoleic acid (C18:2n6) and disclosed the *ADAP2* gene related to phosphatidylinositol bisphosphate binding (GO: 1902936). 

Many other genes were identified in the associated genomic regions; however, their functional annotations did not show a direct relationship with lipids metabolism. The current study identified several genomic regions associated with the fatty acid composition of IMF in rabbits, showing its polygenic nature, since the associated genomic regions did not explain large percentages of the genomic variances. We did not detect genomic regions with mayor effects like those found in pigs [11].

### 3.4. Linkage Disequilibrium Analysis

**Figure 4:**
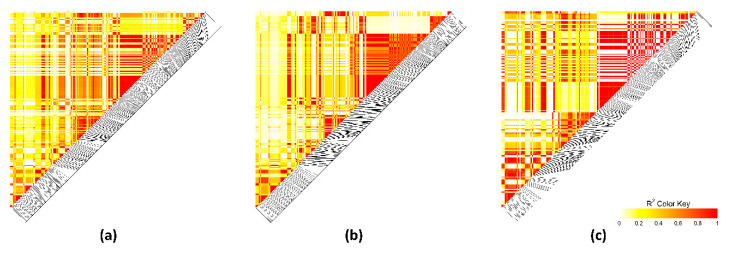
Linkage disequilibrium plot at 34–37.9 Mb on chromosome 1. Colors from white to red indicate r^2^ value. (**a**) All lines. (**b**) High-intramuscular fat (IMF) line. (**c**) Low-IMF line.

**Figure 5:**
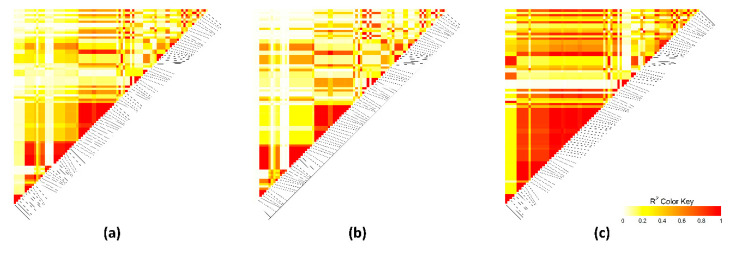
Linkage disequilibrium plot at 148.5–150.2 Mb on chromosome 3. Colors from white to red indicate r^2^ value. (**a**) All lines. (**b**) High-IMF line. (**c**) Low-IMF line.

**Figure 6:**
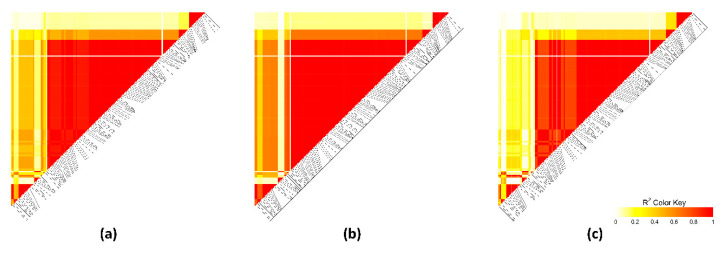
Linkage disequilibrium plot at 46.0–49.2 Mb on chromosome 18. Colors from white to red indicate r^2^ value. (**a**) All lines. (**b**) High-IMF line. (**c**) Low-IMF line

Linkage disequilibrium (LD) was assessed throughout the most relevant extended regions (at 34–37.9 Mb on OCU1, at 148.5–150.2 Mb on OCU3, and at 46.0–49.2 Mb on OCU18). Different LD patterns were detected (Figure 4, Figure 5 and Figure 6). The extended region at OCU1 displayed several short blocks for both high-IMF and low-IMF lines, indicating the presence of several causal variants (QTL). Correlations between SNPs were greater in high-IMF line than in low-IMF line (Figure 4). Besides, the genomic region at 148.5–150.2 Mb on OCU3 displayed different LD patterns between lines. Correlations between SNPs were greater in low-IMF line than in high-IMF line (Figure 5). The QTL at 46.0–49.2 Mb on OCU18 formed a strong block (r^2^ = 1) for both lines, while correlations between SNPs were greater for high-IMF line (Figure 6). Thus, the putative QTLs were exposed to different selection pressures for IMF content. 

### 3.5. Polygenic Nature of the Intramuscular Fatty Acid Composition

Taken together, the present study underlined different genomic regions associated with the fatty acid composition of IMF in rabbits. The results were corroborated having high values of Bayes factors (Figure 1, Figure 2 and Figure 3). The present study highlighted also the polygenic nature of the fatty acid composition and its complexity in rabbits. Similar findings were reported by previous studies for the fatty acid composition in pigs [11,12,13] and cattle [14,16]. To our knowledge, this is the first GWAS for fatty acid composition in rabbits. A pervious study was performed on the IMF content [10]. Comparing the results, *MTMR2* is the only gene that was associated with both IMF content and its composition. In addition, *ACER2* and *PLIN2* genes were associated with the fatty acid composition and were also detected at a selection signature for IMF content [38].

## 4. Conclusions

Association analyses for the fatty acid composition were carried out on two rabbit lines divergently selected for IMF content. Several associated regions were detected. The most relevant regions were found at 34–37.9 Mb on OCU1, at 149.0–149.9 Mb on OCU3, and at 46.0–48.9 Mb on OCU18. The associated regions disclose several genes related to lipid metabolism such as *SCD*, *PLIN2*, *ERLIN1*, and *PPARG*. The main genomic regions in which we found genes related to lipid metabolism were not detected in our previous experiment for IMF. *MTMR2* is the only gene that was associated with both the IMF content and its composition. To our knowledge, this is the first GWAS for fatty acid composition in rabbits. Our study highlighted the polygenic nature of the fatty acid composition as no large percentages of the genomic variances were explained by the associated genomic regions. Further analyses throughout the associated regions would be needed in order to validate their importance.

## Figures and Tables

**Table 1:** Descriptive statistics for fatty acids (% of total fatty acids) of *Longissimus thoracis et lumborum* muscle in rabbits.

Trait	Mean	SD ^1^	CV ^2^ (%)
C14:0	1.26	0.26	20
C16:0	25.7	1.17	4
C18:0	8.34	0.66	8
SFA	36.4	0.94	2
C16:1n7	1.25	0.50	39
C18:1n9	20.3	0.88	4
MUFA	24.4	1.27	5
C18:2n6	27.7	1.18	4
C18:3n3	1.16	0.14	12
C20:4n6	7.45	1.05	14
PUFA	39.2	1.83	4
PUFA/SFA	1.08	0.07	6
MUFA/SFA	0.67	0.03	4
n-6/n-3	17.7	0.97	5

^1^ Standard deviation, ^2^ coefficient of variation, SFA: saturated fatty acids stand for the sum of C12:0, C14:0, C15:0, C16:0, C17:0, C18:0, and C20:0, MUFA: monounsaturated fatty acids stand for the sum of C16:1n7, C18:1n9, C18:1n7, C20:1n11, and C22:1n9, n-3 stand for the sum of C18:3n3, C20:3n3, C20:5n3, C22:5n3, and C22:6n3, n-6 stand for the sum of C18:2n6, C18:3n6, C20:2n6, C20:3n6, C20:4n6, and C22:4n6, PUFA: polyunsaturated fatty acids stand for the sum of C18:3n3, C20:3n3, C20:5n3, C22:5n3, C22:6n3, C18:2n6, C18:3n6, C20:2n6, C20:3n6, C20:4n6, and C22:4n6.

**Table 2:** Genomic regions associated with saturated fatty acids of *Longissimus thoracis et lumborum* muscle in rabbits.

OCU ^1^	Region (Mb) ^2^	Trait	VAR ^3^	PPA ^4^	rSNP ^5^	Number of Genes ^6^	PCG ^7^
1	29.1–29.9	C18:0	0.52	0.55	21	3	-
	34.0–37.9	C14:0	3.54	0.51	38	13	*ACER2, PLIN2*
		C16:0	11.20	0.51	36		
		C18:0	0.87	0.33	1		
		SFA	11.32	0.45	22		
	121.0–121.9	C14:0	0.62	0.69	27	13	*MTMR2*
		C18:0	0.50	0.59	27		
3	149.0–149.9	C14:0	1.11	0.96	19	8	*ENSOCUG00000000157*
		C16:0	3.65	0.98	15		
		C18:0	1.72	0.99	15		
		SFA	0.98	0.51	16		
	154.0–154.9	SFA	0.91	0.39	12	4	*-*
4	67.0–67.9	C18:0	0.71	0.75	14	8	*-*
5	7.2–7.9	C16:0	0.71	0.50	15	1	*-*
6	0–0.9	C16:0	1.33	0.60	12	-	*-*
8	15.0–15.9	SFA	0.52	0.43	25	7	*-*
	21.0–21.9	C18:0	0.57	0.75	69	6	*PIK3C2G, PLCZ1, PLEKHA5*
9	64.0–64.9	C16:0	0.62	0.38	2	10	*NPC1*
		SFA	0.96	0.37	2		
	69.0–69.5	SFA	0.56	0.32	2	4	*-*
10	43.0–43.9	SFA	0.59	0.35	8	3	*-*
13	126.0–126.9	C18:0	0.96	0.87	9	23	*HEYL, MFSD2A, PPT1*
14	82.0–82.9	C18:0	0.51	0.55	18	19	*ADIPOQ*
15	10.0–10.9	C16:0	0.63	0.40	17	11	*LRAT, TLR2*
		SFA	0.63	0.32	16		
18	48.0–48.9	C18:0	0.52	0.52	42	32	*ERLIN1,*
		SFA	0.55	0.41	2		*ENSOCUG00000014801* *ENSOCUG00000001375*

^1^ Rabbit chromosome, ^2^ associated region, ^3^ percentage of the genomic variance explained by the windows belonging to the associated region, ^4^ posterior probability of association, ^5^ number of relevant SNPs with a Bayes factor higher than 10 in the extended region, ^6^ number of protein coding genes in the extended region, ^7^ putative candidate genes related to lipid metabolism retrieved from Database for Annotation, Visualization, and Integrated Discovery (DAVID) database. SFA: saturated fatty acids stand for the sum of C12:0, C14:0, C15:0, C16:0, C17:0, C18:0, and C20:0.

**Table 3:** Genomic regions associated with monounsaturated fatty acids of *Longissimus thoracis et lumborum* muscle in rabbits.

OCU ^1^	Region (Mb) ^2^	Trait	VAR ^3^	PPA ^4^	rSNP ^5^	Number of Genes ^6^	PCG ^7^
1	29.1–29.9	C16:1n7	0.81	0.48	16	3	*-*
	36.0–36.9	C16:1n7	0.92	0.34	3	2	*-*
	121.0–121.9	C18:1n9	0.63	0.34	2	13	*MTMR2*
		MUFA	0.61	0.38	18		
3	149.0–149.9	C18:1n9	1.06	0.64	17	11	*ENSOCUG00000000157*
		MUFA	1.38	0.77	18		
4	67.0–67.9	C18:1n9	0.66	0.41	14	8	*-*
5	7.2–7.9	C16:1n7	0.82	0.61	22	1	*-*
		MUFA	0.99	0.64	22		
6	8–9.9	C16:1n7	0.71	0.64	22	22	*SMG1, GDE1*
		C18:1n9	1.43	0.50	12		
		MUFA	0.67	0.47	10		
8	21.0–21.9	C16:1n7	0.63	0.65	8	6	*PIK3C2G, PLCZ1,*
		C18:1n9	0.72	0.57	4		*PLEKHA5*
		MUFA	1.01	0.66	16		
9	12.0–12.5	C16:1n7	1.05	0.60	17	7	*PPARG*
10	42.2–42.9	MUFA	0.53	0.41	16	1	*-*
13	126.0–126.9	C18:1n9	1.28	0.73	9	23	*HEYL, MFSD2A, PPT1*
		MUFA	0.99	0.69	9		
14	82.0–82.9	C18:1n9	0.58	0.30	7	14	*ADIPOQ*
17	12.0–13.9	C16:1n7	1.55	0.66	35	14	*ALDH1A2, LIPC, MYO1E*
		MUFA	0.81	0.56	36		
18	47.0–48.9	C16:1n7	1.21	0.43	7	35	*GOT1, ERLIN1,*
		C18:1n9	0.83	0.37	2		*ENSOCUG00000014801,*
		MUFA	1.90	0.53	46		ENSOCUG00000001375
21	10.0–10.9	C18:1n9	0.72	0.44	6	2	-

^1^ Rabbit chromosome, ^2^ associated region, ^3^ percentage of the genomic variance explained by the windows belonging to the associated region, ^4^ posterior probability of association, ^5^ number of relevant SNPs with a Bayes factor higher than 10 in the extended region, ^6^ number of protein coding genes in the extended region, ^7^ putative candidate genes related to lipid metabolism retrieved from DAVID database. MUFA: monounsaturated fatty acids stand for the sum of C16:1n7, C18:1n9, C18:1n7, C20:1n11, and C22:1n9.

**Table 4:** Genomic regions associated with polyunsaturated fatty acids of *Longissimus thoracis et lumborum* muscle in rabbits.

OCU ^1^	Region (Mb) ^2^	Trait	Var ^3^	PPA ^4^	rSNPs ^5^	Number of Genes ^6^	PCG ^7^
1	29.1–29.9	PUFA	0.56	0.34	15	3	-
	31–31.9	C18:2n6	1.34	0.27	2	7	*-*
		PUFA	0.6	0.31	16		
	34–37.9	C18:2n6	3.18	0.20	2	13	*ACER2, PLIN2*
		C20:4n6	1.57	0.47	33		
		PUFA	2.41	0.37	11		
2	85.0–85.9	C18:2n6	1.24	0.24	8	3	*-*
3	25.0–26.9	C18:2n6	6.05	0.65	41	6	*FGF1, NR3C1*
	149.0–149.9	C18:2n6	0.68	0.24	1	8	*ENSOCUG00000000157*
		C20:4n6	0.82	0.92	16		
		PUFA	2.25	0.97	15		
5	7.2–7.9	PUFA	0.91	0.66	22	1	*-*
6	0–0.9	PUFA	0.55	0.57	14	-	*-*
	5.0–5.9	C18:2n6	0.61	0.21	8	15	*LITAF, SNX29*
8	21.0–21.9	PUFA	0.78	0.67	14	5	*PIK3C2G, PLCZ1*
	50.0–50.9	C18:2n6	1.06	0.30	-	6	*-*
	55.3–55.9	C18:2n6	4.77	0.30	11	1	*-*
		C18:3n3	0.99	0.38	18		
9	63.0–63.9	C18:2n6	0.61	0.23	1	4	-
	64.0–65.9	PUFA	1.9	0.46	30	16	*NPC1, OSBPL1A*
	115.0–115.9	C18:2n6	1.21	0.38	16	3	*-*
10	43.0–43.9	PUFA	0.58	0.47	17	3	*-*
12	7.0–7.9	C20:4n6	1.48	0.93	10	8	*-*
13	33.0–34.9	C18:2n6	3.42	0.49	13	60	*CRP, DCAF8, FCER1A, PIGM, ATP1A2*
14	156.2–156.9	C18:3n3	0.73	0.74	17	3	*-*
15	10.0–10.9	C18:2n6	2.41	0.39	16	11	*LRAT, TLR2*
		PUFA	0.87	0.53	19		
	70.0–71.9	C18:2n6	3.59	0.50	4	12	*ANXA3, GK2*
16	62.0–62.9	C18:2n6	0.59	0.23	6	9	*HSD11B1*
	71.0–72.9	C18:3n3	1.67	0.43	10	7	*ENSOCUG00000006240*
17	9.0–10.9	C18:2n6	3.87	0.41	21	10	
	12.0–13.9	C18:2n6	3.55	0.46	34	14	*ALDH1A2, LIPC,*
		PUFA	0.84	0.61			*MYO1E*
18	23.0–23.8	C18:2n6	1.18	0.36	17	8	*NRBF2*
19	21.0–21.9	C18:2n6	0.52	0.21	2	15	*ADAP2*
21	10.0–10.9	C18:3n3	0.55	0.68	6	2	*-*

^1^ Rabbit chromosome, ^2^ associated region, ^3^ percentage of the genomic variance explained by the windows belonging to the associated region, ^4^ posterior probability of association, ^5^ number of relevant SNPs with a Bayes factor higher than 10 in the extended region, ^6^ number of the protein coding genes in the extended region. ^7^ putative candidate genes related to lipid metabolism retrieved from DAVID database, PUFA: polyunsaturated fatty acids stand for the sum of C18:3n3, C20:3n3, C20:5n3, C22:5n3, C22:6n3, C18:2n6, C18:3n6, C20:2n6, C20:3n6, C20:4n6, and C22:4n6.

**Table 5:** Genomic regions associated with PUFA/SFA, MUFA/SFA, and n-6/n-3 of *Longissimus thoracis et lumborum* in rabbits.

OCU ^1^	Region (Mb) ^2^	Trait	Var ^3^	PPA ^4^	rSNPs ^5^	Number of Genes ^6^	PCG ^7^
1	29.1–29.9	PUFA/SFA	0.53	0.32	14	3	-
	31.1–31.9	PUFA/SFA	0.59	0.30	11	7	-
	34.0–37.9	PUFA/SFA	2.98	0.38	10	13	*ACER2, PLIN2*
		n-6/n-3	1.45	0.29	17		
3	118.0–118.9	MUFA/SFA	0.58	0.36	1	11	*-*
	149.0–149.9	PUFA/SFA	2.45	0.98	15	8	*ENSOCUG00000000157*
5	7.3–7.9	PUFA/SFA	0.84	0.61	22	1	*-*
6	0.0–0.9	PUFA/SFA	1.09	0.74	14	-	*-*
8	51.0–51.9	n-6/n-3	0.67	0.42	3	7	*-*
	53.0–53.8	MUFA/SFA	0.53	0.28	11	14	*DGKH*
9	64.0–65.9	PUFA/SFA	2.44	0.50	38	16	*NPC1, OSBPL1A*
10	5.0–5.6	MUFA/SFA	0.63	0.37	19	4	*ITGB8*
	43.0–43.9	PUFA/SFA	0.52	0.45	8	3	*-*
14	78.2–78.9	MUFA/SFA	0.52	0.23	5	14	*ATP11B*
15	8.0–8.9	n-6/n-3	0.83	0.48	30	13	*-*
	10.0–10.9	PUFA/SFA	1.60	0.71	18	11	*LRAT, TLR2*
16	62.0–62.9	MUFA/SFA	0.59	0.17	3	9	*HSD11B1*
	71.0–72.9	n-6/n-3	1.04	0.26	10	7	*ENSOCUG00000006240*
18	11.0–11.9	MUFA/SFA	0.86	0.32	3	5	*SAMD8*
	21.0–21.9	MUFA/SFA	0.93	0.52	22	3	*-*
	46.0–48.9	MUFA/SFA	7.91	0.59	54	41	*GOT1, ERLIN1* *ENSOCUG00000014801, ENSOCUG00000001375*

^1^ Rabbit chromosome, ^2^ associated region, ^3^ percentage of the genomic variance explained by the windows belonging to the associated region, ^4^ posterior probability of association, ^5^ number of relevant SNPs with a Bayes factor higher than 10 in the extended region, ^6^ number of the protein coding genes in the extended region, ^7^ putative candidate genes related to lipid metabolism retrieved from DAVID database, SFA: saturated fatty acids stand for the sum of C12:0, C14:0, C15:0, C16:0, C17:0, C18:0, and C20:0, MUFA: monounsaturated fatty acids stand for the sum of C16:1n7, C18:1n9, C18:1n7, C20:1n11, and C22:1n9, n-3 stand for the sum of C18:3n3, C20:3n3, C20:5n3, C22:5n3, and C22:6n3, n-6 stand for the sum of C18:2n6, C18:3n6, C20:2n6, C20:3n6, C20:4n6, and C22:4n6, PUFA: polyunsaturated fatty acids stand for the sum of C18:3n3, C20:3n3, C20:5n3, C22:5n3, C22:6n3, C18:2n6, C18:3n6, C20:2n6, C20:3n6, C20:4n6, C22:4n6.

## Supplementary Materials

The following are available online at https://www.mdpi.com/2076-2615/10/11/2090/s1, Table S1: Relevant validated SNPs associated with the fatty acid composition. Description: ^1^ Validated SNPs with a Bayes factor greater than 50. ^2^ Rabbit chromosome. BF: Bayes factor. SFA: Saturated fatty acids stand for the sum of C12:0, C14:0, C15:0, C16:0, C17:0, C18:0, and C20:0. MUFA: monounsaturated fatty acids stand for the sum of C16:1n7, C18:1n9, C18:1n7, C20:1, and C22:1n9, n-3 stand for the sum of C18:3n3, C20:3n3, C20:5n3, C22:5n3, C22:6n3. n-6 stand for the sum of C18:2n6, C18:3n6, C20:2n6, C20:3n6, C20:4n6, and C22:4n6. PUFA: polyunsaturated fatty acids stand for the sum of C18:3n3, C20:3n3, C20:5n3, C22:5n3, C22:6n3, C18:2n6, C18:3n6, C20:2n6, C20:3n6, C20:4n6, and C22:4n6.
